# Modification of fatty acids composition in erythrocytes lipids in arterial hypertension associated with dyslipidemia

**DOI:** 10.1186/1476-511X-10-18

**Published:** 2011-01-21

**Authors:** Tatyana P Novgorodtseva, Tatyana A Kantur, Yulia K Karaman, Marina V Antonyuk, Natalia V Zhukova

**Affiliations:** 1 Vladivostok Branch of the Far Eastern Center of Physiology and Pathology of Respiration of SB RAMN - Institute of Medical Climatology and Rehabilitative Treatment, Russia; 2 Zhirmunsky Institute of Marine Biology, Far East Branch of Russian Academy of Sciences, Vladivostok, Russia

## Abstract

**Background:**

Modification of fatty acids (FA) composition in erythrocytes lipids as an early indicator of the development of arterial hypertension (AH) and lipid disorders.

**Methods:**

We included 34 patients with arterial hypertension and 11 healthy individuals. Each patient was examined the lipid composition of serum. From erythrocytes by gas chromatography were identified fatty acids. The quantitative composition of the erythrocyte lipids FA in patients with AH presented with saturated and polyunsaturated monoenic acids with carbon chain lengths from C12 to C22.

**Results:**

In all hypertensive patients is disturbed lipid FA composition of erythrocytes. The patients with a normal level of serum lipids revealed deficiency of polyunsaturated acids n6-linoleic (18:2 n6), arachidonic (20:4 n6), dokozatetraenic 14,4% (22:4 n6). The patients with dislipidemia installed more profound restructuring of the lipid matrix of the membrane of erythrocytes. A decrease in polyunsaturated fatty acids family n3: dokozapentaenovoy (22:5 n3), docosahexaenoic PUFA (22:6 n3), the total value of n3 PUFA in 1,3 times was revealed.

**Conclusion:**

Thus, modification of erythrocyte FA are fairly subtle indicator of pathology of lipid metabolism, which manifest themselves much earlier than changes in the lipoprotein of blood plasma.

## Background

Modification of fatty acids composition of lipid matrix of cell membrane has an important role in arterial hypertension (AH) pathogenesis. It's known that changes of the fatty acids (FA) composition, decreasing of quantity of essential polyunsaturated fatty acids (PUFA) may result in membrane microviscosity increasing, proinflammatory eicosanoids synthesis activation and increasing the smooth muscle cells in artery walls sensivity to the influence of vasoconstrictors [1]. Cytosol hypernatremia and hypercalcemia developed in FA forming new ions channels of conductions assist to the function disturbances of friable connective tissue, collagen and elastin increasing both synthesis and secretion. It probably leads to the thickening and decreasing of arteries walls flexibility [2].

Researches devoted to the detailed studying of the modification of FA in erythrocytes lipids in arterial hypertension don't create general image of typical changes of FA composition in this category of patients which connected to the variety of associated disorders (obesity, resistance to the insulin, diabetes mellitus).

Arterial hypertension and dyslipidemia (DLP) - most important and commonly present risk factors [3-7], with prevalence of 50-80% [8-11] according to the different authors. Among nontreated patients with AH the frequency of DLP is 40%, on the other hand almost one half of patients with increased level of total cholesterol (TC) has systolic and diastolic hypertension [12-15]. The confirmation of adverse prognostic significance of hypercholesterolemia was obtained by results of the multicenter studies such as MRFIT (Multiple Risk Factor Intervention Trial) and Seven Countries Study. According to these studies the growth of absolute and relative characteristics of mortality from cardiovascular disease in direct dependence of TC level [16-19] were demonstrated on large populations.

That's why the great interest is that immersed studying of changing of spectrum of FA in erythrocytes lipids in patients with arterial hypertension associated with dyslipidemia.

## Materials and methods

The research was performed according to the principles of Helsinki Declaration (2000), with 34 patients with diagnosed AH 1-2 stage, 11 male and 13 female (average age - 49,21 ± 1,7). The control group consisted of 11 almost healthy persons.

The criteria for the inclusion into the study was diagnosed AH 1-2 stage. During diagnosis of AH practical recommendations of European Society of Cardiology (ESC) and European Society of Hypertension (ESH) [20] were used.

The criteria for exclusion were AH with 3 stage, secondary hypertension, associated with the pathology of urinary system (urolithiasis, acute and chronic pyelonephritis, polycystic kidney disease), endocrine system (diabetes, hyperthyroidism, Cushing disease), as well as concomitant diseases of the cardiovascular system (coronary heart disease, stroke, myocardial infarction, cardiac arhythmias).

The serum lipid spectrum was studied by the content of cholesterol, triglycerides (TG), high density lipoprotein cholesterol (HDL) in serum with biochemical analyzer Photometer FM 750 (Germany), using sets of firm "Olvex" (Russia). Results were expressed in mmol/liter.

The concentration of low density lipoproteins cholesterol (LDL) and very low (VLDL) density were calculated by the formula: LDL = total cholesterol - (HDL + VLDL), VLDL = TG/2,2, the results were expressed in mmol/liter. Atherogenicity index (AI) was calculated by the formula: AI = (TC - HDL)/HDL [21].

The extraction of lipids from erythrocytes was performed by a modified method of Bligh and Dyer [22]. Gas-liquid chromatography of FA methyl ethers was performed on gas-liquid chromatograph "Shimadzu-9A" (Japan) with a flame ionization detector. Methyl ethers of erythrocytes FA lipids were obtained by the method of Carreau and Dyubak [23] and purified by thin-layer chromatography. FA methyl ethers were analyzed by capillary columns. The Chromaton N-AW-DMCS 0,100-0,125 was used as carrier, 5% Silar-5 CP, FFAP - as the liquid phase. The evaporation temperature was 245°C. The separation temperature was 210°C. Helium was the gas-carrier with linear velocity - 20 cm/sec. The identification was performed using standard mixtures of FA and the values of the equivalent chain length [24,25]. Quantitative calculations were performed using the standard software of data processing systems Chromatopak-CR 3A. Results were expressed in relative % of total FA. Unsaturation index (UsI) was calculated as the sum of the products of double bonds in each of the FA with its relative percentage.

Statistical data processing was performed using the methods of descriptive statistics: the arithmetic mean, the standard error of arithmetic mean (M ± m), the criteria for significant differences (t) Student [26].

## Results

Analysis of clinical and laboratory parameters allowed to divide all patients with AH depending on the state of lipid metabolism into 2 groups: 1 group - 16 patients with normolipidemia, 2 group - 18 patients with dyslipidemia. The criteria for dyslipidemia were levels of total cholesterol (TC) >5.0 mmol/l, LDL cholesterol (LDL) >3.0 mmol/l, triglycerides (TG) >1.77 mmol/l, high density lipoprotein cholesterol (CH HDL) <1.0 mmol/l, atherogenic index (AI) >3.5 a.u. [20]. Table 1 demonstrates the lipidogramm of the observed persons. Level of serum lipids in patients in 1 group was normal. Patients in 2 groups have an increased concentration of total cholesterol, TG, LDL, VLDL, and IA, associated with the reduced level of HDL (Table 1).

**Table 1:** Clinical and biochemical characteristics of patients with arterial hypertension, M ± m

Parameters	Control group, n = 11	Patients with AH, n = 24	
		1 group (without DLP), n = 16	2 group (with DLP), n = 18
Systolic arterial pressure, mmHg.	105 ± 2	***141 ± 2	***141 ± 1
Diastolic arterial pressure, mmHg	65 ± 2	***87 ± 1	***88 ± 1
TC, mmol/l	4,44 ± 0,21	4,33 ± 0,06	**5,16 ± 0,13***
ТG, mmol/l	0,68 ± 0,07	**0,92 ± 0,04	***1,79 ± 0,14***
HDL, mmol/l	1,41 ± 0,11	1,29 ± 0,02	***0,97 ± 0,05***
LDL, mmol/l	2,72 ± 0,22	2,63 ± 0,06	***4,00 ± 0,06***
VLDL, mmol/l	0,31 ± 0,03	*0,4 ± 0,01	***0,65 ± 0,01***
AI, a.u.	2,27 ± 0,05	2,45 ± 0,08	***4,35 ± 0,09***

Note: tabl. 1, 2 (*) - Statistically significant differences linked to the control group are on the left, - for the 1group are on the right: * - p < 0.05, ** - p < 0.01, *** - p < 0,001.

The quantitative composition of the erythrocyte lipids FA in patients with AH presented with saturated and polyunsaturated monoenic acids with carbon chain lengths from C12 to C22 (Table 1). The table doesn't contain some minor components of the FA which content does not exceed 0,1%. As follows from Table 2, the FA composition in patients with AH without DLP demonstrated increased proportion of saturated and monoenic FA and reducing of polyenic FA.

**Table 2:** The content of FA in erythrocyte lipids in patients with AH, % of total FA sum M ± m

Fatty acids	Control group, n = 11	Patients with AH, n = 34	
		
		1 group (without DLP), n = 16	1 group (with DLP), n = 16
14:0	0,65 ± 0,04	0,76 ± 0,06	***0,41 ± 0,04***
15:0	0,28 ± 0,02	0,28 ± 0,02	*0,41 ± 0,05*
16:0	23,37 ± 0,32	22,70 ± 0,72	***25,90 ± 0,42***
16:1n7	0,80 ± 0,03	***0,5 ± 0,05	0,85 ± 0,09**
17:0	0,45 ± 0,04	***0,72 ± 0,02	0,42 ± 0,01***
17:1n9	trace	0,20 ± 0,01	trace
18:0	17,23 ± 0,29	***25,2 ± 0,18	17,03 ± 0,39***
18:1n9	13,25 ± 0,55	*14,85 ± 0,48	**15,02 ± 0,32**
18:1n7	1,38 ± 0,10	1,40 ± 0,02	1,60 ± 0,06**
18:2n6	13,74 ± 0,53	**11,60 ± 0,45	14,5 ± 0,2***
18:3n6	trace	1,28 ± 0,16	2,10 ± 0,54
18:4n3	trace	0,27 ± 0,01	trace
20:0	0,18 ± 0,02	***0,31 ± 0,02	trace
20:1	0,28 ± 0,04	***0,40 ± 0,01	trace
20:2n6	0,28 ± 0,02	0,34 ± 0,03	0,25 ± 0,02*
20:3n6	1,16 ± 0,05	1,10 ± 0,06	*1,34 ± 0,05**
20:3n9	0,48 ± 0,05	***0,86 ± 0,06	***0,88 ± 0,18
20:4n6	11,13 ± 0,24	***10,5 ± 0,2	***13,18 ± 0,18***
20:5n3	1,08 ± 0,11	1,16 ± 0,04	1,32 ± 0,10
22:4n6	2,15 ± 0,11	*1,84 ± 0,10	1,89 ± 0,13
22:5n6	0,2 ± 0,01	0,28 ± 0,01	trace
22:5n3	2,42 ± 0,16	2,22 ± 0,17	**1,81 ± 0,08*
22:6n3	5,8 ± 0,3	6,48 ± 0,35	**4,18 ± 0,40***
Sum 20-22n6	16,35 ± 0,52	15,30 ± 0,42	**14,38 ± 0,41
Sum 20-22n3	9,2 ± 0,3	9,6 ± 0,8	*7,70 ± 0,55*
20-22n3/20-22n6	0,58 ± 0,03	0,65 ± 0,05	0,50 ± 0,03*
Sum n6	30,1 ± 0,6	**27,01 ± 0,66	30,2 ± 0,4***
Sum n3	9,98 ± 0,78	10,0 ± 0,5	*7,5 ± 0,7**
n3/n6	0,31 ± 0,01	**0,37 ± 0,02	*0,26 ± 0,02**
22:6n3/22:5n3	2,4 ± 0,1	***2,95 ± 0,12	2,26 ± 0,15***
20:4n6/22:6n3	2,11 ± 0,12	1,83 ± 0,12	*2,53 ± 0,13***
20:4n6/20:3n6	10,41 ± 0,40	10,72 ± 0,55	***8,54 ± 0,31***
20:4n6/20:5n3	13,01 ± 0,99	**9,25 ± 0,52	***8,01 ± 0,42
Unsaturation index	155,98 ± 1,33	**141,32 ± 4,38	157,9 ± 1,9**

Note: tabl. (*) - Statistically significant differences linked to the control group are on the left, - for the 1 group are on the right: * - p < 0.05, ** - p < 0.01,

*** - p < 0,001.

There was a substantial increased level of margarinic (17:0) to 60%, stearic (18:0) to 46.3%, eykozanoic (20:0) to 72.2%, oleic (18:1 n9) to 12% and appearance of geptadekamonoenic (17:1 n9) FA.

Such changes in monoenic acids composition make the membrane more susceptible to oxidation while oleic acid, being active endogenous invader of the active oxygen forms, becomes a source of cytotoxic products of lipid peroxidation, initiates the processes of membrane destruction [1]. The reduction of the proportion of fatty acids family n6 - linoleic (18:2 n6), arachidonic (20:4 n6), dokozatetraenic 14,4% (22:4 n6) was set, which naturally resulted in the decrease in the total index of unsaturated FA n6. Changes in the quantity of PUFA n3 was not revealed. Unsaturation index decreased due to the lack of polyene n6.

An increased level of Mead acid 20:3 n9 to 79% was revealed, which compensatory synthesis occurs in case of deficit of the polyunsaturated acids family n6 and n3. The increase of the proportion of Mead acid and marked increasing of the level of oleic acid (18:1 n9) declare active synthesis of substrates for the formation of prostacyclines and thromboxanes, which have vasoconstrictor properties [2,27].

Changes in the FA composition may be the result of metabolic disturbances in the transformations. Reduction ratio 20:4 n6/20: 5n3 detected in 1group patients reflected the disturbance of FA synthesis - the forerunner of eicosanoics of the different series. This state of FA metabolism can lead to increased production of oksilipins 2nd and 4th series demonstrating vasoconstrictor, aggregation properties [1,28].

Comparative analysis of parameters between the groups revealed some differences in changes of single acids in patients with DLP. Most pronounced changes in quantitative amount of saturated FA were established. An accumulation of pentadekanoic (15:0) and palmitic (16:0) acids were revealed. As in 1group, patients with AH associated with dyslipidemia have a high content of oleic acid and Mead acid.

Changes in PUFA composition are more pronounced, especially with acids n3 structure. PUFA family n3deficiency was confirmed with significant decrease of the proportion of dokozapentaenoic (22:5 n3) and docosahexaenoic PUFA (22:6 n3). There was a reduction of the total value of n3 PUFA in patients with DLP in 1.3 times in comparison with 1 group.

A distinctive parameter of patients with AH associated with DLP was rising of forerunner of eicosanoids with vasoconstrictor effects - arachidonic acid (20:4 n6) to 26%. This is a sign of polyunsaturated acids n3 lack. The imbalance in the composition of PUFA n3 and n6 is reflected in the decreasing ratio n3/n6. The 2 group patients have the accumulation of saturated and polyunsaturated n6 FA with deficit of PUFA n3 in the cytomembrane which indicates on the decreasing of the fluidity of cell lipid bilayer, an activity of membrane bound enzymes and inhibition of binding the ligands with the receptor [1,27,29].

In respect that one of the reasons for modification of the FA is a disturbance of its metabolism, their rates of the transformations were analyzed. The analysis of parameters of the FA metabolic enzymes activity revealed the reduction of the ratio 20:4 n6/20: 3n6, which indicating the inhibition of eicosatrienoic acid metabolism. Discovered changes in the ratio 20:4 n6/20: 5n3 and 20:4 n6/22: 6n3 involve an imbalance in eicosanoids cycle in the direction of increasing the oksilipins synthesis with marked vasoconstrictor (thromboxane A2) and proinflammatory (leukotriene B4) properties [1,27].

The results indicate a disturbance of the FA erythrocytes lipids in patients with AH regardless to the presence or absence of hyperlipidemia. A characteristic feature of the modification of erythrocytes lipids FA composition in patients with normolipidemia is increased proportion of margarinic, stearic, eicosanoic saturated FA, Mead acid with low values for the polyunsaturated acids n6 - linoleic and arachidonic.

It is known that the development of hypercholesterolemia preceded by blockade of cells active absorption of essential polyunsaturated FA [1,2,27]. An indicator of pathology of the active ligand-receptor transport of FA is PUFA n3 and n6 deficiency. PUFA deficit modifies the fluidity of annular phospholipids in the cells plasmatic membrane, contributes to the dysfunction of the bilayer integral proteins (ion pumps, receptors, transport and signaling systems), which, being in an anhydrous environment, change their conformation and function. At the same time with developing the blockade of income polyenic acids into the cell and changes in receptor system, active transport into the cell of saturated FA of VLDL breaks down by apoE/V-100 endocytosis. The consequence of this process is compensatory activation of adipocytes triglyceride hydrolysis and increased elimination of saturated FA into the blood flow. In this way conditions for the TG increasing in the blood and the development of hypertriglyceridemia are created. Therefore pathological changes in the composition of membrane lipids arising by the reason of the breach of FA active transport leading to the formation of AH and also become inevitable independent factors for development of hyperlipidemia, which was confirmed by our studies [1,30].

It was shown that patients with AH associated with hypercholesterolemia and hypertriglyceridemia revealed a deeper restructuring of the erythrocytes membrane lipid matrix. The increased proportion of the pentadekanoic, palmitic and arachidonic acid were demonstrated. A distinctive feature of the modification of erythrocytes lipids FA in patients with AH associated with DLP was deficiency of PUFA n3. The observed prevalence of saturated FA proportion in the erythrocytes with simultaneous lack of polyunsaturated acids may be the evidence of transport pathology with a predominance of cells passive FA absorption. The integral parameters of the cells impaired absorption both of saturated FA and PUFA are low levels of HDL and high levels of TG and cholesterol in the blood serum [1,2,27]. Cells endogenous deficiency of n3 PUFA leads to the changes of the plasmatic membranes physicochemical properties, activation of eicosanoids synthesis with proinflammatory and vasoconstriction activity, the formation of systemic inflammatory syndrome and conditions for the atherogenesis [1].

Thus, structural and functional changes in erythrocyte membranes in hypertension developed as a part of the common systemic disorders of lipid metabolism. Defects of receptor-mediated mechanism for the FA capture induce the formation of AH. The increase in the disorganization of the lipid components of the cell membrane causes the development of hyperlipidemia and progression of hypertension. Modification of erythrocyte FA is rather subtle indicator of pathology of lipid metabolism manifested much earlier than lipoproteins changes in the blood plasma. These findings clearly demonstrate the important role of fatty acids in the pathogenesis of cardiovascular diseases.

## Competing interests

The authors declare that they have no competing interests.

## Authors' contributions

TK, YK and NZ participated in the design of the study, performed the statistical analysis and drafted the manuscript, contributed to acquisition of data and its interpretation. MA contributed to conception and design of the statistical analysis. NT conceived of the study, participated in its design, coordination and helped to draft the manuscript. All authors read and approved the manuscript.
